# Comparison of the Aroma Profiles of Intermediate Wheatgrass and Wheat Bread Crusts

**DOI:** 10.3390/molecules24132484

**Published:** 2019-07-06

**Authors:** Laurianne Paravisini, Kelsey A. Sneddon, Devin G. Peterson

**Affiliations:** 1 Department of Food Science and Technology, 2015 Fyffe Road, The Ohio State University, Columbus, OH 43210, USA; 2 Department of Food Science and Nutrition, 145 Food Science and Nutrition Building, 1334 Eckles Avenue, University of Minnesota, St. Paul, MN 55108, USA

**Keywords:** whole wheat, intermediate wheatgrass, bread, aroma, Maillard reaction, *Thinopyrum intermedium*

## Abstract

The aroma profiles of bread crusts made from intermediate wheatgrass (*Thinopyrum intermedium*) and whole wheat (*Triticum aestivum*) flours were compared. Based on gas chromatography/mass spectrometry/olfactometry analysis, twenty-four odorants were identified and further quantified. The concentrations of seventeen compounds were significantly different between intermediate wheatgrass and whole wheat bread crusts, of which sixteen compounds were higher in the whole wheat sample. The aroma profiles of the bread samples were subsequently characterized using sensory descriptive analysis (DA) and indicated that the roasted attribute was perceived at a significantly higher intensity in the whole wheat sample due to a greater amount of Maillard reaction compounds. Alternatively, bran and green notes were perceived at higher intensities in the intermediate wheatgrass sample, however they were not attributed to the presence of specific compounds but rather to a change in the aroma composition. Aroma recombination DA of the whole wheat and intermediate wheatgrass aroma models was similar to the original aroma profiles of the bread samples, demonstrating the sensory relevance of the identified odorants.

## 1. Introduction

Common wheat (*Triticum aestivum*) is among the world’s most traded commodities and is projected to increase in trade by almost 16% over the next ten years to reach 212 million metric tons by 2027/2028 [1]. Intermediate wheatgrass (IWG, *Thinopyrum intermedium*) has been identified as one of the most promising candidates as a wheat alternative due to its perennial growth trait which is beneficial for sustainable agricultural practices [2]. It presents a rich nutritional profile and is high in dietary fiber [3]. However, little is known regarding the flavor performance of IWG when incorporated in foodstuffs. A small-scale sensory study showed that muffins, cookies and cake made with various amounts of IWG were acceptable to consumers [4]. However, the impact of IWG on flavor in comparison to common wheat is not adequately understood. Flavor, along with other factors such as convenience and cost, are key factors that influence food choice [5,6]. Consequently, an improved understanding of how IWG impacts the flavor attributes of food products is needed.

Bread is a staple in many countries and constitutes the main source of whole grain intake in the North American diet [7]. More than 500 volatile compounds have been reported in bread with the majority of the studies focusing on bread made from refined wheat flour [8]. In an effort to promote whole wheat bread consumption, previous work characterized whole wheat aroma and taste compounds to understand mechanisms of flavor formation in wheat bread [9,10]. Moskowitz et al. (2012) [8] compared the aroma of bread made from refined and whole wheat flours. This work demonstrated the impact of compounds present at higher concentration in the refined bread, such as 2-acetyl-1-pyrroline, 2-acetylthiazoline and 2-phenylethanol, on overall aroma intensity. 

The pathways of flavor formation in common wheat bread have been well characterized. Fermentation and enzymatic reactions primarily occur during bread making and initiate the breakdown of lipids and oligosaccharides to form small molecular weight compounds, sensory-active themselves or important transient flavor precursors [11]. During baking, thermal reactions including lipid oxidation, caramelization and the Maillard reaction have been identified as main sources of flavor generation. These pathways are greatly influenced by processing conditions and grain composition. For example, thermal reactions are favored in the crust, which is exposed to higher temperatures than the crumb—this results in the formation of higher levels of N-heterocycle compounds [12]. The composition of the grain is known to impact flavor formation due to changes in precursors, which is influenced by both genetic and environmental factors. Rye and wheat flours have been compared in regard to their impact on the generation of bread aroma [13,14]. The potent and key bread odorant 2-acetyl-1-pyrroline, for example, was found at 20-fold higher amount in bread crust made from wheat in comparison to rye, likely due to favorable reaction conditions of higher proline and saccharide content in the wheat flour. The oligosaccharide distribution in wheat and rye is significantly different and also greatly varies depending on the agricultural practices [15]. Similarly, IWG grain has compositional differences with common wheat. Total dietary fiber content and structures of oligosaccharides have been characterized [3,16], and would be expected to affect flavor generation in foodstuffs.

The overall goal of this work is to characterize the aroma profiles of IWG and whole wheat breads. Aroma-active compounds in the crusts were identified using Gas Chromatography/Mass Spectrometry/Olfactometry (GC/MS/O) and further quantified. Sensory descriptive analysis of the bread samples and recombination models was then performed to define aroma differences as impacted by flour type and to validate the sensory relevance of the identified compounds.

## 2. Results and Discussion

### 2.1. Identification and Quantification of Volatile Aroma Compounds of Whole Wheat and IWG Bread Using GC/MS/O

Aroma profiles of bread crusts formulated with either whole wheat or IWG flour were characterized using GC/MS/O analysis. Overall, both crusts showed similar volatile profiles and were composed of aroma compounds previously reported in wheat bread such as ketones, alcohols, esters and heterocycle compounds (Table 1). Twenty-four compounds were identified and quantified in whole wheat and IWG crust samples. Significant differences were observed in the quantitative profiles of the crusts for 17 compounds out of 24, among which 16 were present in higher levels in the whole wheat sample.

Nine of the compounds previously reported as Maillard reaction products were identified, including pyrazines and α-acetyl-*N*-heterocycles (Table 1). 2-Acetylfuran, 2-ethyl-3,5-dimethylpyrazine and 2-ethyl-3,6-dimethylpyrazine, associated with yeasty, roasted and chocolate notes, respectively, showed the highest fold-change with concentrations approximately 20-fold higher in the whole wheat bread compared to the IWG sample. The two pyrazines were detected below 1 μg/kg in the IWG bread, i.e., approximately at their odor thresholds in water [17], whereas their concentrations were at levels of 16 μg/kg in whole wheat bread, suggesting their important contribution to crust aroma. 2-Acetylfuran, described as having yeast and fermented odor notes, was quantified below its odor threshold in both breads (<10,000 μg/kg) but its formation was significantly favored in the whole wheat sample. Furanoid compounds can exhibit a wide range of odor properties from green to sweet-brown notes depending on their concentration and are important flavor compounds and/or flavor intermediates. Their significant impact on the sensory properties of thermally treated food has been widely demonstrated [18,19]. 

The generation of 2-acetyl-1-pyrroline and 2,3-diethyl-5-methylpyrazine was also significantly impacted by grain-type and was present at 2- to 3-fold higher levels in the whole wheat sample (versus the IWG sample). Specifically, 2-acetyl-1-pyrroline has been widely reported as a major contributor to bread aroma and exhibits an extremely low odor threshold (0.1 μg/L in water) [20]. In this study, 2-acetyl-1-pyrroline was quantified between 4.0 (IWG) and 9.2 (wheat) μg/kg, or at 400- and 10,000-fold above the odor threshold, respectively and thus likely contributed to the different characteristic aroma of the bread crust. In wheat bread, 2-acetyl-1-pyrroline formation is thought to occur from the reaction between 1-pyrroline, a Strecker degradation product of proline, and methylglyoxal, a reactive carbonyl species originating from reducing sugars [21]. 

The Maillard compound 2,3-diethyl-5-methylpyrazine was described by the panelists during GC/O as having earthy, roasted and vegetal odor notes with significant differences in concentration between IWG and whole wheat, from 1.1 and 3.1 μg/kg, respectively (Table 1). This pyrazine, also called hazelnut pyrazine, has been widely reported in food but only two studies have reported its occurrence in wheat bread crust [14,22] and the impact on the sensory properties of bread has not been studied. In beef, 2,3-diethyl-5-methylpyrazine is known to be generated from the thermal reaction between reducing sugars (glucose and fructose) and alanine with methylglyoxal as a key sugar fragment reactive intermediate [23]. IWG grain has been reported to have a significantly higher amount of alanine compared to common wheat [4]. This suggests that alanine is not the limiting factor for pyrazine formation in bread. Others have reported 2,3-diethyl-5-methylpyrazine formation from glucose and glycine in model systems with a central role of the reactive intermediate species methylglyoxal for pyrazine formation [24].

Both diacetyl and methional are known contributing odorants in food and can originate from both the Maillard reaction and yeast metabolism [8]. In the bread samples, both compounds were formed in greater amounts in the wheat sample, about 5-fold higher for methional and 2-fold for diacetyl.

Overall, the Maillard-related aroma composition in the wheat bread was approximately 4-fold higher than in the IWG sample with the total amount of Maillard compounds equaling approximately 2000 and 455 μg/kg, respectively. This noted difference in aroma generation can be related to the compositional difference between wheat and IWG flour samples. For example, IWG grain contains present a higher amount of proteins, about 20% vs. 14% in wheat, whereas wheat contains a slightly higher amount of carbohydrates, about 69% vs. 62% in IWG [4]. Changes in the sugar: the N-containing ratio can have a significant impact on the Maillard formation pathways by modulating the generation of reactive intermediates and ultimately modifying the volatile aroma fraction [25,26].

Additionally, the total dietary fiber content in IWG grain is higher than in whole wheat, approximately 17% of dry content vs. 3% [16], and would be expected to result in a higher amount of hydroxycinnamic acids, which are known to interact with Maillard reaction intermediates and suppress the formation of potent aroma compounds [10].

A parallel can be drawn with gluten-free bread which has been reported to lack the generation of Maillard aroma compounds such as pyrazines and 2-acetyl-1-pyrroline [27,28]. IWG gluten protein structures are substantially different to wheat gluten proteins, as IWG protein consists of mainly α and γ-glutenins and some low molecular weight glutenins [29]. However, wheat protein also contains a high molecular weight fraction of glutenins (lacking in IWG), which is known to be critical to the gluten network and give desirable texture and consistency to common wheat bread [16]. The absence of high molecular weight glutenins in IWG may have impacted the flavor formation in IWG bread crust (Table 1) and suggests the possible role of high molecular weight glutenins on Maillard reaction pathways and in particular the formation of pyrazines. IWG has also been reported to have differences in composition of the oligosaccharide fraction compared to common wheat. Structural differences in arabinoxylans, major hemi cellulosic constituents of cereal grains, have been demonstrated [3]. In green coffee beans, the formation of furans was not only associated with low molecular weight sugars such as sucrose but also with arabinogalactan pyrolysis [30]. The noted changes in the aroma composition of wheat and IWG bread crust (Table 1) therefore could have been related to the structure of the oligosaccharides, that are unique to each cereal species.

Only one Maillard reaction compound, 2-acetyl-2-thiazoline, was present at a higher level in the IWG bread crust, at 16 μg/kg versus 9.8 μg/kg in the wheat sample. 2-Acetyl-2-thiazoline is a potent aroma compound, known for its typical popcorn odor note and very low odor threshold, 1 μg/kg in water [31]. This compound has been previously identified in refined wheat bread crust extract [32], however its role on the aroma attributes of bread has not been demonstrated. This compound originates from the reaction between cysteine and reducing sugars [31] which aligns with prior findings that show a higher amount of cysteine in the IWG about 1.4-fold higher than wheat [4].

Five aldehydes associated with green, oxidized and grain odor notes were identified in the bread samples (Table 1). Aldehydes are typical lipid oxidation products that can be unwanted in food products (at elevated levels) but are also associated with the characteristic aroma of whole wheat bread [11]. The concentration of the three unsaturated aldehydes, (*E*)-2-nonenal, (*E*,*Z*)-2,6-nonadienal and (*E*,*Z*)-2,6-nonadienal, was significantly impacted by the grain-type and present at 2- to 4-fold higher levels in the wheat sample. Interestingly, IWG has a significantly higher lipid content, up to 20% higher, than common wheat [4] and yet, the formation of lipid oxidation products was unfavored. This indicates that the amount of precursor is not a determinant factor and suggests differences in enzyme activity such as lipoxygenase or inherent factors to the grain (higher antioxidant content, such as phenolics) that impact the formation of aldehydes through modulation of lipid oxidation pathways.

Seven esters, alcohols and ketones associated with floral, green and fruity notes were also identified (Table 1) and are thought to arise from fermentation pathways. The concentration of five compounds was slightly impacted and present at 1.5- to 2-fold higher amount in the wheat versus the IWG sample. Typically, a higher amount of yeast would promote the formation of these compounds [11]. However, in the current study, the two bread samples were formulated with the same amount of yeast, indicating that other parameters inherent to the grain are modifying the fermentation pathways.

Finally, three phenolic compounds, *p*-vinylguaiacol (or 4-methoxy-2-vinylphenol), salicylaldehyde and guaiacol, were identified in the bread samples (Table 1). Only *p*-vinylguaiacol was significantly impacted by the grain-type and exhibited a concentration 5-fold higher in the wheat sample. This compound is a known degradation product from ferulic acid generated during thermal and/or enzymatic reactions [33]. As previously mentioned, a higher total amount of dietary fiber is present in IWG in comparison to wheat [16]. Ferulic acid is one of the most abundant hydroxycinnamic acids present in wheat and IWG; however, the differences between the two grains in regard to that particular compound have not been reported. Work in beer demonstrated that the correlation between the amounts of ferulic acid and *p*-vinylguaiacol was not evident; however, processing conditions and decarboxylase enzyme activity can impact product formation [33], suggesting that other factors, such as initial phenolic amount, can modulate the formation of volatile phenols in bread.

The final two compounds identified (Table 1), salicylaldehyde and guaiacol, have been previously reported in pseudo-cereals such as buckwheat [34] and no significant differences in concentration were detected between the IWG and common wheat samples.

### 2.2. Sensory Evaluation of Bread Crusts and Aroma Recombination Models

**Figure 1:**
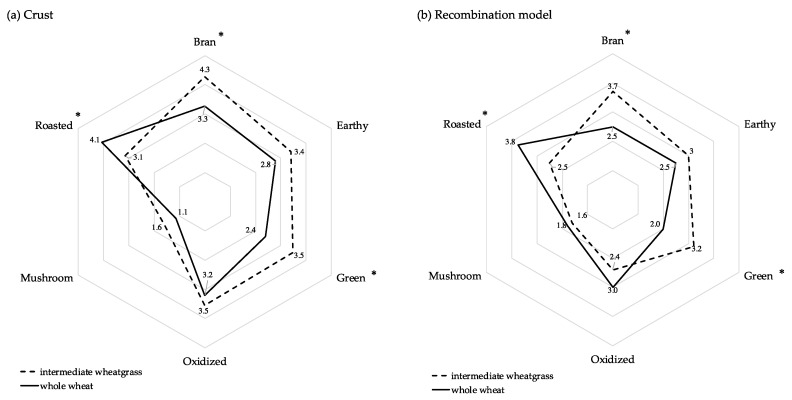
Aroma profiles from sensory descriptive analysis of whole wheat and intermediate wheatgrass (IWG) bread crusts (**a**) and aroma recombination models (**b**). * Indicates significant effect of sample on aroma intensity (*p* < 0.05).

Descriptive analyses of the wheat and IWG bread crusts and corresponding aroma recombination models were carried out to characterize the aroma profiles of the bread samples and to evaluate the sensory relevance of identified compounds. The primary aroma attributes in the bread samples were defined as bran, roasted, earthy, green, oxidized and mushroom and are shown in Figure 1a; the corresponding aroma profiles for the recombination models are shown in Figure 1b. No significant differences for all six aroma attributes were reported between the bread sample (Figure 1a) and the corresponding recombination model (Figure 1b), indicating that the identified compounds sufficiently characterized the aroma attributes.

Overall, wheat and IWG bread crust had the same primary aroma attributes that differed in intensity (Figure 1a). Roasted was the most intense attribute in the wheat sample rated at 4.1, significantly higher than the IWG crusts rated at 3.1. Roasted is a characteristic attribute of fresh bread crust that has been widely attributed to the presence of Maillard reaction compounds [13]. This is in accordance with the current study that demonstrated 4-fold more Maillard reaction products in the wheat sample compared to the IWG sample. The thermally generated odorant structure α-acetyl-N-heterocycles (such as 2-acetyl-1-pyrroline and 2-acetyl-2-thiazoline) is thought to elicit common odor traits, typical of bread aroma and sweet-brown notes [31,35]. Although this odor property has been demonstrated to be valid when these compounds are evaluated individually, the impact of an individual compound (odor) to the overall perception of bread aroma (as a complex mixture) has been subject to controversy and is not adequately defined [32]. The present findings are non-conclusive towards the role of α-acetyl-*N*-heterocycles in crust aroma, and specifically the roasted note. As previously noted, 2-acetyl-2-thiazoline was the only compound significantly higher in the IWG sample and yet this sample exhibits the lowest roasted intensity when evaluated by sensory analysis. These results emphasize the need to complement the analytical results with sensory evaluation in order to transfer the findings into more sensory relevant outcomes. For example, the roasted aroma attribute of bread could be the result of a combination of compounds that elicit this trait when present in a specific balance. In the current study, the IWG bread aroma fraction is significantly lacking in most of the aroma compounds identified that ultimately resulted in a shift in the overall sensory profile.

Conversely, bran and green attributes were rated significantly higher in the IWG with intensities of 4.3 and 3.5, respectively, versus 3.3 and 2.4 in the wheat sample. This finding may be due to the lower levels of Maillard compounds that decreased the roasted note, and consequently enhanced the perception or intensity of bran and green attributes. The intensities of earthy and oxidized averaged around 3, indicating an important contribution of these attributes to the overall bread crust aroma profile, however these attributes were not rated as significantly different between the wheat and IWG samples.

In summary, the analytical results were in alignment with the sensory findings showing significant differences between the aroma composition and sensory attributes of bread crusts made from wheat and IWG. Generally, the wheat bread samples contained higher levels of aroma compounds that resulted in a different overall sensory profile. The aroma drivers of bread liking have not been well defined; however, some specific traits are known to be associated with lower consumption of whole grain products such as green and oxidized [36]. This study demonstrated the dominance of these aroma attributes for IWG and can potentially hinder the development of highly accepted products in the future. Overall, this work provides a basis to optimize breeding and processing strategies for more palatable whole grain foods.

## 3. Materials and Methods

### 3.1. Chemicals

The following compounds, 2-methylpropanal, 2,3-butanedione, 3-methylbutanal, methional, 2-acetylfuran, 1-octen-3-one, 1-octen-3-ol, (*E*)-2-nonenal, (*E*,*Z*)-2,6-nonadienal, 2-acetylpyrazine, 2-acetyl-2-thiazoline, 2-ethyl-3,5(and 6)-dimethylpyrazine, (*E,E*)-2,4-decadienal, 1-hexanol, 2-phenylethanol, salicylaldehyde, p-vinylguaiacol, ethyl nonanoate, and ethyl octanoate were purchased from Sigma Aldrich (St. Louis, MO, USA). 2-Ethyl-3,5-dimethylpyrazine and 2-ethyl-3,6-dimethylpyrazine were separated using preparative GC and [^2^H_3_]-2-Acetyl-1-pyrroline and [^2^H_4_]-2-acetyl-2-thiazoline were synthesized as previously described [37]. 3-Methyl-1-butanol, guaiacol, and 2-methyl-3-heptanone were purchased from Tokyo Chemical Industry (TCI; Portland, OR, USA). 2,3-Diethyl-5-methylpyrazine, butylated hydroxytoluene (BHT), ethanol (200 proof) and dichloromethane were purchased from Fisher Scientific (Pittsburgh, PA, USA). Sodium bicarbonate and anhydrous sodium sulfate were purchased from Avantor (Center Valley, PA, USA).

### 3.2. Preparation of Bread Samples

King Arthur Flour premium 100% hard red whole wheat unbleached flour was purchased from the local market. Intermediate wheatgrass (IWG) groats were provided by the Land Institute (Salina, KS, USA). King Arthur Flour premium 100% hard red whole wheat unbleached flour (obtained from the local market) was used for the preparation of whole wheat bread samples, and IWG (IWG) groats, provided by the Land Institute (Salina, KS, USA), were ground in a whole grain flour cyclone sample mill (UDY, Fort Collins, CO, USA) equipped with a 0.25 mm screen for the preparation of IWG bread samples. Fleischmann’s Dry Active Yeast (ConAgra, Chicago, IL, USA), Crisco (The J.M. Smucker Co., Orville, OH, USA) vegetable shortening, Crystal Sugar (American Crystal Sugar Company, Moorhead, MN, USA) granulated sugar, and Morton Salt (Chicago, IL, USA) iodized salt were purchased from the local market. Bread samples were prepared using an adjusted AACC method 10-10B (AACC, 1995). Briefly, sugar (72 g), salt (18 g), and yeast (24 g), were mixed with 150 g of warm (39 °C) distilled water. Flour (1200 g of either wheat or IWG) was added to a mixing bowl and was combined with the sugar, salt and yeast solution as well as vegetable shortening (36 g) that had been melted at 60 °C. The total amount of distilled water was then brought to 873.6 g. The dough was mixed with a bread hook for seven minutes using a 10-speed stand mixer (KitchenAid Artisan KSM150PSWH, St. Joseph, MI, USA). The fermentation, punching schedule, and proofing were as dictated in the AACC 10-10B method through the use of a fermentation cabinet/proofing oven (Baxter PW2E, East Orting, WA, USA). Loaves were then placed in a rotary oven, primed with 1 L of water, at 215 °C for 17 min. The loaves were immediately removed from the pans and prepped for solvent extraction.

### 3.3. Aroma Extraction Using Solvent-Assisted Flavor Evaporation (SAFE)

Immediately after baking, the bread crust was separated from the crumb, frozen with liquid nitrogen, and ground using a food processor. Crust (625 g) was extracted with 1.25 L of dichloromethane (DCM) spiked with 8 μg of 2-methyl-3-heptanone as an internal standard, and 8 μg of BHT to serve as an antioxidant. The extraction was performed under a blanket of nitrogen gas and agitation using an orbital shaking table (Thermo Scientific MaxQ™ HP Tabletop Orbital Shaker, Waltham, MA, USA) at room temperature for 15 h. The solvent was removed by filtering through glass wool, and filter paper (Whatman Filter Paper 4, GE, Pittsburgh, PA, USA). Liquid–liquid extraction was repeated twice. Organic extracts were pooled and dried with anhydrous sodium sulfate, and then distilled down to approximately 100 mL using a Vigreux distillation column (60 cm) and a water bath maintained at 35 °C. The solvent extract was then isolated by Solvent-Assisted Flavor Evaporation (SAFE) [38]. The volatile isolate recovered by SAFE was neutralized by washing three times with a 1:1 volume of 0.5 M sodium bicarbonate and once with a half volume equivalent of saturated sodium chloride. The isolate was dried with anhydrous sodium sulfate, distilled down to approximately 5 mL using a Vigreux distillation column (60 cm) and finally concentrated to approximately 1 mL under a flow of nitrogen. Extraction was carried out on three biological replicates.

### 3.4. Identification of Aroma Compounds Using Gas Chromatography/Mass Spectrometry/Olfactometry (GC/MS/O)

GC/MS/O analyses were performed using an Agilent GC G1530A (Agilent Technologies, Santa Clara, CA, USA) coupled with a Hewlett Packard 6890 mass spectrometer detector (MSD) (Hewlett Packard, Palo Alto, CA, USA), and an olfactometric port (Hillesheim, Waghäusel, DE, Germany). The effluent was split 1:1 after separation, between the MSD and the olfactometric port. Purified air was bubbled through distilled water and introduced in the nose cone at 20 mL/min to reduce nasal dehydration. One microliter of the volatile extract was injected using splitless injection. Inlet temperature was set at 250 °C, helium carrier gas set to a constant flow of 1.5 mL/min. For identification purposes, samples were run on two columns of different polarities; an Agilent HP-5 (30 m × 0.25 mm × 0.25 μm film thickness) and an Agilent DB-Wax (30 m × 0.25 mm × 0.25 μm film thickness). GC conditions for the HP-5 column were as follows: 40 °C was initially held for 1 min and then ramped at a rate of 3 °C/min until 150 °C, followed by 30 °C/min ramp to 250 °C and held for 5 min. GC conditions for the DB-Wax were as follows: GC oven initial temperature was 40 °C and held for 1 min, ramped at 3 °C/min to 180 °C, then ramped at 30 °C/min to 240°C and held for 5 min. MS conditions were as follows: MS transfer line was set at 250 °C, source temperature 150 °C, mass range *m/z* 29–350 at 2.35 scans/sec operated in electron ionization (EI) mode. Compounds were positively identified using mass spectra, odor descriptors, and linear retention index (LRI) in comparison to the match with authentic compounds and database (NIST 2.2). LRI values were calculated with an *n*-alkane ladder from C_6_ to C_25_.

For GC/O analyses, each sample was serially diluted to 1/2 volume in dichloromethane and analyzed until no further aromas were detected. Each dilution was analyzed in duplicate by three panelists (one female, two males). The largest dilution at which each aroma was detected, by at least one panelist, was defined as its FD value. All compounds smelled at a dilution higher or equal to 16 were selected.

### 3.5. Quantification of Aroma Compounds Using Stable Isotope Dilution Assay and Standard Addition

2-Acetyl-2-thiazoline and 2-acetyl-1-pyrroline were quantified using stable isotopes dilution assay and their deuterated homologues [^2^H_4_] and [^2^H_3_], respectively. Known amounts of deuterated 2-acetyl-2-thiazoline and 2-acetyl-1-pyrroline were added to the bread crust 20 minutes before extraction. The extracts were then prepared in triplicate as previously described. Analyses were carried out using the same GC conditions as previously described and with the MS operating in positive chemical ionization (PCI) mode using methane as a reagent gas under single ion monitoring (SIM) mode for maximum sensitivity. Quantification was based on a five-point calibration curve that showed good linearity, r^2^ ≥ 0.99.

The remaining compounds were quantified using dynamic headspace and standard addition method. The crust (10 g) was placed in 50 mL of deionized water and spiked with known amounts of aroma compounds. The sample was then placed in a 250 mL jacketed 3-way purge and trap chamber maintained at 60 °C. High purity nitrogen was purged through the sample at a rate of 25 mL/min for 10 min onto an adsorbent trap packed with 180 mg of Tenax TA (Gerstel, Mülheim an der Ruhr, Germany). The trap was further dried purged for 20 min. The volatile compounds were desorbed from the trap at 240 °C for 4 min under a flow rate of 50 mL/min. Compounds were cryofocused on the CIS maintained at −100 °C, then desorbed at 250 °C for 3 min and then introduced onto the column under splitless mode for 0.5 min. GC/MS was carried out as previously described. MS was operating in SIM mode. Selected ions are given in Table 1. Each addition was carried out in triplicate for each bread crust. Five-point standard addition curves were constructed for all compounds in each bread crust that showed good linearity, r^2^ ≥ 0.95.

### 3.6. Sensory Descriptive Analysis of Bread Samples and Recombination Models

Sensory descriptive analysis was carried out on wheat and IWG crusts, and their respective aroma recombination model systems. The panel consisted of 6 trained participants (two females and four males, ages 24 to 32) that had previously received training in descriptive analysis techniques and had at least one year of experience performing as a sensory panelist. In addition, participants were screened for their ability to differentiate the aroma of the bread crusts. Lexicon development and panelist training occurred over 16 1-hr sessions. The attributes, descriptors, and reference samples chosen by the panel are shown in Table 2.

Wheat and IWG bread crust samples for sensory evaluation were prepared as previously described. Prior to analysis, each sample (6 g bread crust) was placed in a 60-mL amber bottle at room temperature for 2 h to allow for equilibration. Aroma recombination models were prepared by adding the determined concentration of aroma compounds in ethanol and then the mixture was sprayed onto filter paper. The solvent was allowed to evaporate for 3 min, and then the paper was inserted into a 60-mL amber bottle and placed at room temperature for 2 h to allow for equilibration. Samples were labeled with 3-digit codes and presented in a randomized order. Panelists were asked to evaluate the aroma orthonasally and rate the intensities of the attributes on a 13 categorized box scale (0 = not present; 1 = threshold; 5 = weak; 9 = moderate; 13 = strong). Data were collected in triplicate (two samples per session for a total of three session) using Compusense Cloud Software (Compusense Inc., Guelph, ON, Canada).

### 3.7. Data Analysis

For the sensory data, a three-way ANOVA with fixed effect model (sample, panelist (fixed), replicate and interactions) and all two-way interactions was carried out. Replicate effect and panelist*sample interactions were not significant for the selected attributes, showing consistency of the data and good agreement among the panel towards the sensory concept. Aroma compound concentrations were compared between wheat and IWG samples using t-test. All analyses were carried out using JMP Pro 13.1.0 (SAS Institute Inc., Carry, NC, USA).

## Figures and Tables

**Table 1:** Concentration of aroma compounds identified in whole wheat and intermediate wheatgrass bread crust.

Retention Index		MS-SIM *(m*/*z)*	Compound Name	Odor Description ^1^	Average ± SD ^2^ (µg/kg) *	
DB-Wax	HP-5				Whole Wheat	Intermediate Wheatgrass
<1000	<600	72	2-methylpropanal	malty, grain	3456 ± 515	2786 ± 88
<1000	<600	43	Diacetyl	butter	1876 ± 81	1298 ± 174 *
<1000	655	86	3-methylbutanal	pungent, grain	1839 ± 171	1475 ± 572
1216	730	70	3-methylbutanol	musty, grain, fruity	10,103 ± 887	5319 ± 1209 *
1360	870	56	1-hexanol	bready, fruity	660 ± 56	691 ± 134
1451	907	104	Methional	potato, earthy, roasted	522 ± 29	110 ± 13 *
1508	913	95	2-acetylfuran	yeasty, fermented	1355 ± 46	81.9 ± 5.7 *
1333	924	112	2-acetyl-1-pyrroline	crust, bread, corn chip	9.2 ± 0.6	4.0 ± 0.2 *
1308	980	55	1-octen-3-one	green, metallic, sharp	9.8 ± 1.5	4.2 ± 0. 5 *
1462	986	57	1-octen-3-ol	green, herbs	90.2 ± 8.4	116± 24
1693	1020	121	salicylaldehyde	pungent, grain, cooked, medicinal	1.2 ± 0.1	1.1 ± 0.3
1625	1028	43, 122	2-acetylpyrazine	baked, bread	593 ± 40	351± 134
1457	1090	135	2-ethyl-3,6-dimethylpyrazine	roasted, chocolate, earthy	16.4 ± 1.6	0.71 ± 0.04 *
1465	1094	135	2-ethyl-3,5-dimethylpyrazine	sweet, burnt, roasted	11.5 ± 0.7	0.66 ± 0.21 *
1855	1096	109	Guaiacol	sweet, cream	13.1 ± 2.8	11.9 ± 0.7
1756	1114	87	2-acetyl-2-thiazoline	crust, corn chip	9.8 ± 0.7	15.5 ± 0.86 *
1934	1132	91	2-phenylethanol	floral, sweet, yeasty	4225 ± 514	2551 ± 666 *
1499	1155	150	2,3-diethyl-5-methyl-pyrazine	earthy, roasted, vegetal	3.3 ± 0.3	1.1 ± 0.1 *
1584	1157	41, 80	(*E*,*Z*)-2,6-nonadienal	green, fresh	13.6 ± 2.1	7.1 ± 1.8 *
1538	1167	55	(*E*)-2-nonenal	green, oil, beany	2714 ± 280	1478 ± 165 *
1442	1204	88	ethyl octanoate	green, wine	169 ± 28	50.3 ± 4.1 *
2224	1281	150	*p*-vinylguaiacol	cooked, sweet, pasta	4611 ± 693	911 ± 246 *
1542	1295	88	ethyl nonanoate	tropical, fruity	32.4 ± 5.6	10.8 ± 2.7 *
1806	1316	81	(*E*,*E*)-2,4-decadienal	cooked oil	856 ± 151	194 ± 24 *

^1^ Odor description for GC/MS/O analysis of bread solvent extract. ^2^ Standard deviation of three biological replicates. * Significantly different according to t-test (*p*-value < 0.05).

**Table 2:** Definition of sensory attributes and references used for descriptive analysis.

Attributes	Descriptors	References
Earthy	Soil, dirt, earthy	Canned chickpeas (La Preferida)
Roasted	Slightly nutty, roasted, browned	Roasted sunflower nuts (Good Sense)
Oxidized	Musty, wet cardboard, oil	Steel cut oats (Bob’s Red Mill)
Green	Green, herb, grassy	Whole grain buckwheat flour (Bob’s Red Mill)
Bran	Sweetened cereal, grain, processed cereal	Bran cereal (General Mills Fiber One Original)
Mushroom	Raw mushroom	Baby bella mushrooms (from local market)
